# PCBP1 regulates the transcription and alternative splicing of metastasis‑related genes and pathways in hepatocellular carcinoma

**DOI:** 10.1038/s41598-021-02642-z

**Published:** 2021-12-02

**Authors:** Shuai Huang, Kai Luo, Li Jiang, Xu-Dong Zhang, Ying-Hao Lv, Ren-Feng Li

**Affiliations:** 1 Departments of Hepatobiliary Surgery, The First Affiliated Hospital of Zhengzhou University, Zhengzhou, 450052 Henan People’s Republic of China; 2 Departments of Radiology, The First Affiliated Hospital of Zhengzhou University, Zhengzhou, 450052 Henan People’s Republic of China

**Keywords:** Cancer genomics, Data mining, Data processing, Genome informatics

## Abstract

PCBP1 is a multifunctional RNA-binding protein (RBP) expressed in most human cells and is involved in posttranscriptional gene regulation. PCBP1 regulates the alternative splicing, translation and RNA stability of many cancer-related genes and has been identified as a potential tumour suppressor gene. PCBP1 inhibits the invasion of hepatocellular carcinoma (HCC) cells, but there are few studies on the specific regulatory target and mechanism of RBPs in HCC, and it is unclear whether PCBP1 plays a role in tumour metastasis as a splicing factor. We analysed the regulation of gene expression by PCBP1 at the transcriptional level. We obtained and analysed PCBP1-knockdown RNA-seq data and eCLIP-seq data of PCBP1 in HepG2 cells and found that PCBP1 widely regulates the alternative splicing and expression of genes enriched in cancer-related pathways, including extracellular matrix, cell adhesion, small molecule metabolic process and apoptosis. We validated five regulated alternative splicing events affected by PCBP1 using RT-qPCR and found that there was a significant difference in the expression of APOC1 and SPHK1 between tumour and normal tissues. In this study, we provided convincing evidence that human PCBP1 profoundly regulates the splicing of genes associated with tumour metastasis. These findings provide new insight into potential markers or therapeutic targets for HCC treatment.

## Introduction

In 2020, hepatocellular carcinoma (HCC) was the sixth most prevalent malignant tumour and the third leading cause of cancer-related death in the world according to GLOBOCAN 2020 (Global Cancer Burden Assessment released by the International Agency for Research on Cancer). The number of new cases of liver cancer worldwide in 2020 was 905,677, with 830,180 deaths; HCC accounts for 75–85% of all liver cancer cases^1^. The epidemiological risk factors for HCC are chronic infection of hepatitis B virus (HBV) or hepatitis C virus (HCV), aflatoxin, alcohol abuse and type 2 diabetes^2–4^. HCC has the worst survival rate of all cancers in China, mainly because it is difficult to diagnose early. Advanced HCC has a high degree of malignancy, rapid progression, low sensitivity to chemotherapeutic drugs, high toxicity, and poor curative effects. Although chemotherapy or multikinase inhibitors play a positive role in the survival of patients, the prognosis is still very poor. HCC is a heterogeneous and convoluted disease caused by genetic and epigenetic mutations in many tumour suppressor genes (TSGs) and oncogenes, as well as disorders of coding or noncoding genes. At present, there is an urgent need to deeply analyse the pathogenesis of HCC and explore molecular markers and therapeutic targets to improve the therapeutic effect^5,6^.

As key molecules in genome regulation, RNA-binding proteins (RBPs) can interact extensively with DNA at the chromatin level and can also interact with RNA at the molecular level to form a ribonucleoprotein (RNP) complex^7^. RBPs cover or expose coding and noncoding regions at different stages of the mRNA functional cycle, control the maturation and fate of target RNA substrates and regulate many aspects of gene expression^8,9^.

The PCBP1 gene, which encodes an RBP, is located on human chromosome 2 (2p13-12), which is described as a tumour suppressor gene region. PCBP1 is a multifunctional linker protein expressed in most human cells and is involved in the regulation of many biological processes. Some previous studies focused on PCBP1 and transformed epithelial cells, emphasizing its role in tumour metastasis and progression, while others suggested that PCBP1 is a tumour suppressor^10–14^. In related studies of HCC, PCBP1 was closely related to the epithelial-mesenchymal transition (EMT) process of hepatoma carcinoma cells. It can bind to circulating RNA, playing an important role in the metastasis of HCC, and reverses the effect of circulating RNA on the migration ability of tumour cells. Similarly, another study confirmed that there was a negative correlation between PCBP1 and the expression of its upstream lncRNA PCBP1-AS1^15^. In a previous study, researchers confirmed that PCBP1 has a significant regulatory effect on the alternative splicing of CD44 in HepG2 cells and SMMC7721 cells. After knocking down endogenous PCBP1, both HepG2 cells and SMMC7721 cells showed enhanced invasion ability, suggesting that PCBP1 inhibited tumour invasion and metastasis. In particular, in HepG2 cells, PCBP1 inhibited CD44 subtype exon inclusion by combining with the splicing silencing element in the exon and inhibited the invasion of HepG2 cells through this mechanism^16^. PCBP1 inhibits the invasion of HCC cells and regulates the splicing of large numbers of metastasis-related genes, but there are few studies on the specific regulatory target and mechanism of PCBP1 in HCC. This project analysed the RNA sequencing (RNA-seq) data and enhanced crosslinking and immunoprecipitation sequencing (eCLIP-seq) data of PCBP1-knockdown HepG2 cells and obtained the differentially expressed genes (DEGs) and significant differential alternative splicing (AS) events (ASEs), as well as the characteristics of PCBP1-bound RNA. Combined with the results of eCLIP, the regulation of target genes by PCBP1 was analysed. We found that genes whose transcriptional level is regulated by PCBP1 and regulated alternative splicing events (RASEs) affected by PCBP1 are associated with tumour metastasis. This finding lays a foundation for a follow-up study of the function of PCBP1 in HCC.

## Results

### PCBP1 overexpression changes the gene expression profiles of HepG2 cells

We downloaded original RNA-seq reads of sh-PCBP1 cells (the PCBP1 gene was silenced by short-hairpin RNA (shRNA)) and control cells (the cells were transfected with negative control shRNA) detected by RNA-seq from the ENCODE database^9^. Sample descriptions are presented in Supplementary Table S1. After trimming the sequence adaptors and filtering out low-quality reads, we acquired an average of 69.1 million clean paired-end reads for each sample. These read results were mapped to the human genome, by which we obtained an average of approximately 62.5 million uniquely mapped read pairs for each sample (Supplementary Table S2).

**Figure 1:**
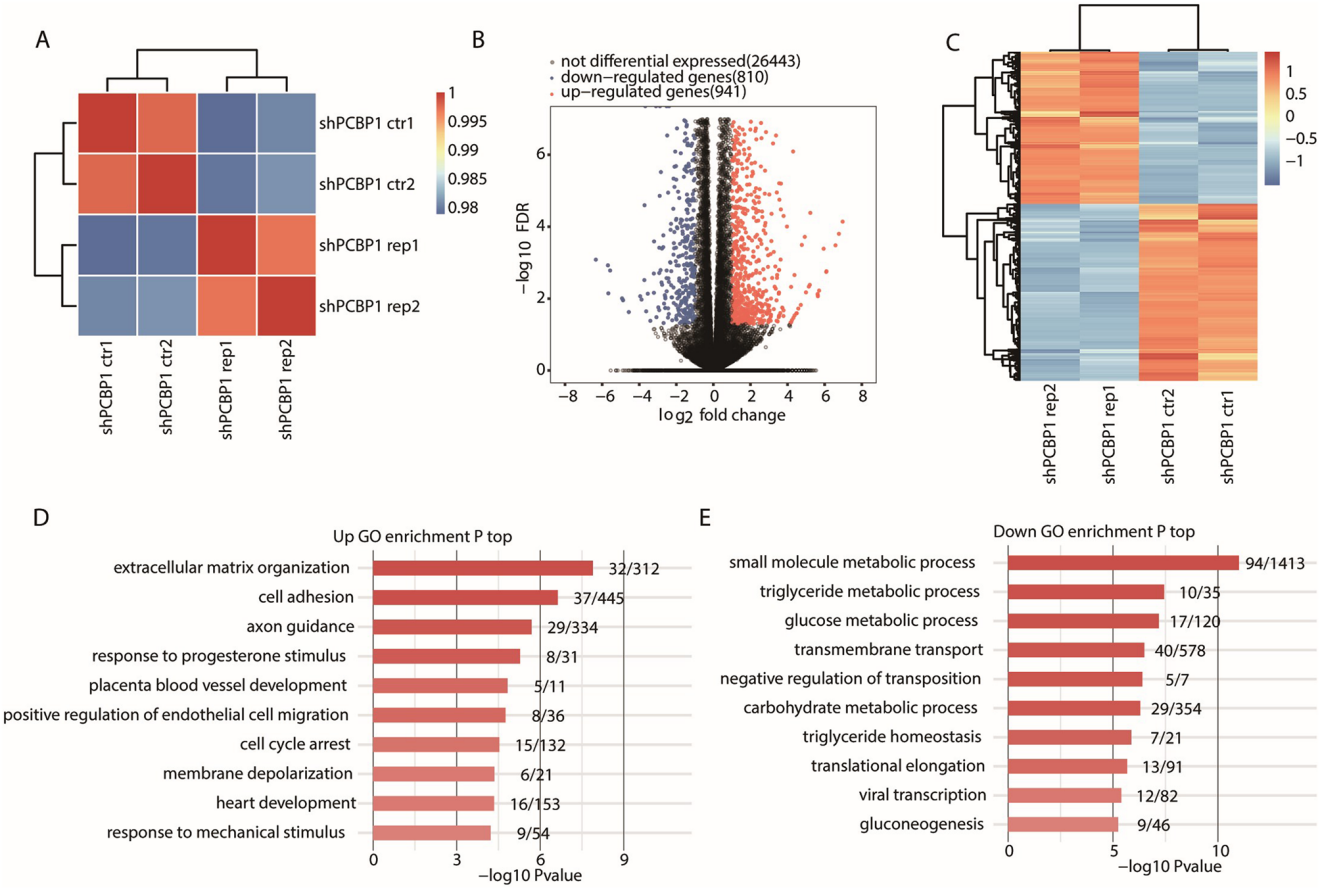
Transcriptome-wide analysis revealed abnormal gene expression after knockdown PCBP1 in HepG2 cells. (**A**) Hierarchical clustering heat map showing correlation between PCBP1-knockdown (shPCBP1 rep1/2) and control (shPCBP1 ctr1/2) samples based on FPKM value of all expression genes. (**B**) Volcano plot showing all differential expressed genes (DEGs) between PCBP1-knockdown and control samples using DESeq2. FDR ≤ 0.05 and FC (fold change) ≥ 2 or ≤ 0.5. (**C**) Heatmap showing expression profile of all significant DEGs between PCBP1-knockdown and control samples. (**D**) Bar plot showing the top10 most enriched GO biological process terms of the up-regulated DEGs. (**E**) Bar plot showing the top10 most enriched GO biological process terms of the downregulated DEGs. PCBP1, Poly C Binding Protein 1; FPKM, fragments per kilobase of exon per million; DEGs, differentially expressed genes; FDR, false discovery rate; FC, fold change.

For correlation analysis of the gene expression level, we focused the analysis on each pair of samples. As revealed in Fig. 1A, there was a significant correlation between sh-PCBP1 cells and control cells. Although there were only two replicate groups, the two biological replicates were significantly related. This means that DEGs has good repeatability.

When examining the DEGs among the samples, the thresholds for significant differences were set to fold change (FC) ≥ 2 or ≤ 0.5 and false discover rate (FDR) < 0.05. A volcano plot revealed that 1751 DEGs were significantly related to PCBP1 knockdown.

We identified 941 upregulated genes and 810 downregulated genes among the DEGs (Fig. 1B), which indicated that PCBP1 knockdown globally regulates the level of gene expression in HepG2 cells. Moreover, hierarchical cluster analysis of normalized DEG fragments per kilobase of exon per million fragments mapped (FPKM) values presented a clear separation of the sh-PCBP1 and control samples and revealed the high consistency of the two replicate data sets (Fig. 1C). These results revealed that PCBP1 knockdown globally influenced the transcript expression level of a set of genes.

### The overexpression of PCBP1 affects the expression of a set of genes that promotes HCC metastasis

We performed functional annotation of all 941 DEGs using Gene Ontology (GO) and Kyoto Encyclopedia of Genes and Genomes (KEGG) enrichment analyses. We listed the top 10 GO terms of the biological process category in sh-PCBP1 cells, including upregulated and downregulated genes, in Fig. 1D and E (details in Supplementary Table S3). The upregulated genes of sh-PCBP1 cells were mainly enriched in extracellular matrix (ECM) organization and cell adhesion (Fig. 1D). ECM organization changes the biochemical and physical properties of the tumour microenvironment, which influences tumour migration and cancer cell polarity and signalling^17–19^. A decrease in intercellular contacts would generate appropriate conditions for the migration of tumour cells. The downregulated genes were mostly associated with small molecule metabolic processes, triglyceride metabolic processes, transmembrane transport and glucose metabolic processes (Fig. 1E). Previous studies have shown that glucose metabolism, particularly glycolysis, is of great significance in tumour development and metastasis^20–22^. Abnormal lipid metabolism is associated with the growth and metastatic cascade of HCC and other tumours^23,24^.

According to the KEGG results (details in Supplementary Table S4), the upregulated gene sets were mainly enriched in osteoclast differentiation, the TNF signalling pathway, the NF-kappa B signalling pathway and ECM–receptor interaction (Supplementary Fig. S1A). The downregulated gene sets were significantly enriched in metabolic pathways, carbon metabolism and biosynthesis of amino acids (Supplementary Fig. S1B). These results revealed that PCBP1 is of great importance in regulating ECM, cell adhesion, positive regulation of endothelial cell migration, cell cycle arrest, ECM–receptor interaction and cell adhesion molecules (CAMs), which are directly associated with tumour cellular proliferation, adhesion and metastasis.

### Analysis of potential PCBP1-regulated ASEs and genes in small molecule metabolic processes

To study the regulatory effect of PCBP1 on AS, PCBP1-affected ASEs were analysed through the use of transcriptional sequencing data from HepG2 cells. For AS analysis, we aligned RNA-seq data samples to unique mapped reads on the reference genome. We detected 248,393 exons in total (Table 1), accounting for up to 67.62% of all annotated exons in the reference genome. The splice junctions of all samples were then analysed by employing HISAT2 (v2.2.1, http://daehwankimlab.github.io/hisat2/) software. Through this process, we identified 160,162 known splice junctions (Known_Junction) and 271,827 novel splice junctions (Novel_Junction) (Table 2). ABLas was used to perform the statistical analysis process of all RASEs. The detection results are presented in Table 3. A total of 55,238 ASEs were detected, including 39,532 nonannotated novel ASEs.

**Table 1:** Exon detection results in RNA-seq data.

Sample	Detected_exon	Annotated exon	Ratio
shPCBP1_ctr1	219839	367321	59.85%
shPCBP1_ctr2	215393	367321	58.64%
shPCBP1_rep1	224432	367321	61.10%
shPCBP1_rep2	225108	367321	61.28%
Total	248393	367321	67.62%

*RNA-seq* RNA sequencing, *PCBP1* poly C binding protein 1.

**Table 2:** Splicing junction analysis of samples from RNA-seq data.

Sample	All_Junction	Novel_Junction	Known_Junction
shPCBP1_ctr1	245860	103473	142387
shPCBP1_ctr2	225313	85936	139377
shPCBP1_rep1	247425	103534	143891
shPCBP1_rep2	248299	104101	144198
Total	431989	271827	160162

*RNA-seq* RNA sequencing, *PCBP1* poly C binding protein 1.

**Table 3:** All AS events detected from all samples.

Sample	Novel AS (NAS)	All AS	NAS%
shPCBP1_ctr1	18380	29682	61.92%
shPCBP1_ctr2	16753	27009	62.03%
shPCBP1_rep1	20237	31723	63.79%
shPCBP1_rep2	20544	32072	64.06%
Total	39532	55238	71.57%

*AS* alternative splicing, *PCBP1* poly C binding protein 1.

**Figure 2:**
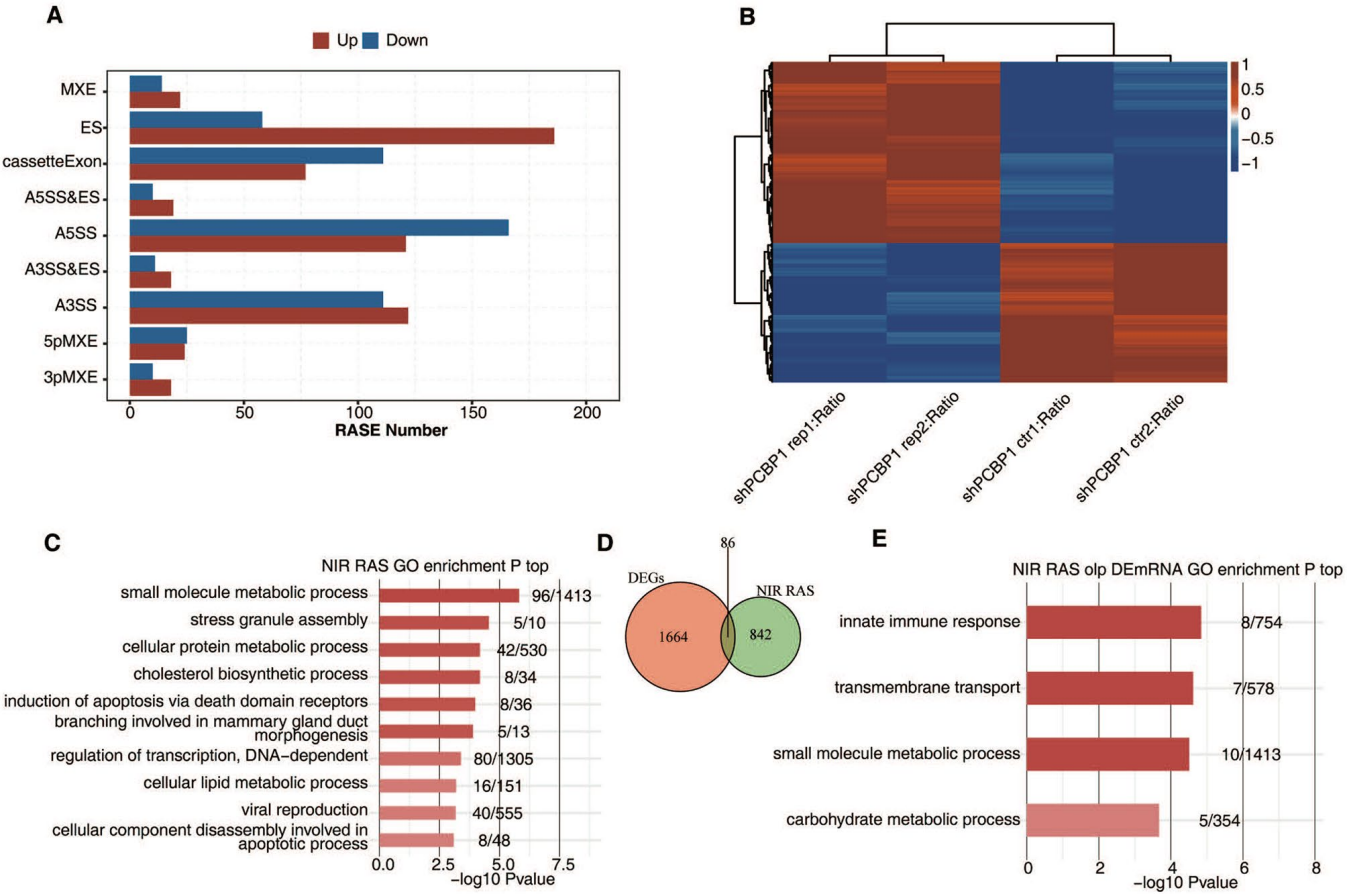
The occurrence of variable splicing events with significant differences suggested the regulatory role of PCBP1 in HepG2 cells. (**A**) The bar plot showing the number of all significant regulated alternative splicing events (RASEs). X-axis: RASE number. Y-axis: the different types of AS events. (**B**) Hierarchical clustering heatmap of all significant NIR RASEs based on PSI. AS filtered should have detectable splice junctions in all samples and at least 80% samples should have >  = 10 splice junction supporting reads. (**C**) Bar plot exhibited the most enriched GO biological process results of the NIR regulated alternative splicing genes (RASGs). (**D**) Venn diagram showing the overlap genes number of NIR RASGs and DEGs. (**E**) Bar plot exhibited the most enriched GO biological process results of overlap genes in D. PCBP1, Poly C Binding Protein 1; NIR RASEs, non-intron-retention regulated alternative splicing events; PSI, percent spliced in; AS, alternative splicing; GO, Gene Ontology; DEGs, differentially expressed genes.

As revealed in Fig. 2A, we detected 2,076 RASEs, and the details are presented in Table 4. Of the RASE types, there were 233 alternative 3′ splice site (A3SS) events, 287 alternative 5′ splice site (A5SS) events, 244 exon skipping (ES) events, 188 cassette exon events and 953 intron retention events. When PCBP1 was knocked down, the proportion of ES events was increased. The results indicated that PCBP1 could promote the selection of weak exons in the entire genome. These data revealed that PCBP1 has a significant impact on AS at the genome level in HepG2 cells. The above results suggested that PCBP1 regulates ASEs in HepG2 cells.

**Table 4:** Classification of all RASE events between sample groups.

Sample	shPCBP1_vs_ctrl	shPCBP1_vs_ctrl
Type	Up	Down
3pMXE	18	10
5pMXE	24	25
A3SS	122	111
A3SS&ES	18	11
A5SS	121	166
A5SS&ES	19	10
ES	186	58
IntronR	757	196
MXE	22	14
cassetteExon	77	111
Total	1364	712

*RASE* regulated alternative splicing events, *PCBP1* poly C binding protein 1, *3pMXE* mutually exclusive 3′UTRs, *5pMXE* mutually exclusive 5′UTRs, *A3SS* alternative 3′splice site, *ES* exon skipping, *A5SS* alternative 5′splice site.

We plotted a clustering heatmap to show the splicing ratio of AS genes. The red region of the experimental group represents the upregulated genes, while the red region of the control group indicates the downregulated genes. The results showed that the differences in regulated AS genes (RASGs) caused by PCBP1 knockdown were highly consistent between the two groups (Fig. 2B). GO enrichment analyses were performed on the differential RASGs, and the top 10 terms are presented in Fig. 2C. The GO enrichment results revealed that PCBP1-regulated ASEs were mainly enriched in small molecule metabolic processes, cellular protein metabolic processes and cellular lipid metabolic processes as well as apoptotic processes. In a variety of tumour models, changes in the expression levels of small molecules lead to tumour degeneration, which is related to remodelling of the tumour microenvironment and tumorigenesis^25–27^.

We carried out an integrated analysis on RASGs and DEGs. The results indicate that 86 genes had significant differences in terms of the AS level and expression level (Fig. 2D). GO functional enrichment was performed on 82 DEGs. The results showed that these genes were mainly enriched in innate immune response, transmembrane transport, small molecule metabolic process and metabolic process (Fig. 2E). In summary, these results showed that PCBP1 undoubtedly regulated ASEs involved in pathways associated with small molecule metabolic process genes, which are strongly associated with tumorigenesis.

### PCBP1 eCLIP-seq reads are preferentially enriched in 3′UTR and CDS regions

The eCLIP-seq approach was used to identify PCBP1-interacting transcripts in HepG2 cells. We downloaded three PCBP1 eCLIP-seq samples from the ENCODE database and divided them into 3 groups (PCBP1_IP_1, PCBP1_IP_2, and PCBP1_Ctrl) (https://www.encodeproject.org/experiments/ENCSR635FRH/). In this project, adapter (AGATCGGAAGAGC) removal, quality filtering, PCR duplicate read elimination and other processes were carried out for the downloaded data to acquire high-quality data, and the clean reads were compared to the reference genome by STAR software (v2.7.3a, https://github.com/alexdobin/STAR).

eCLIP-seq sample descriptions are presented in Supplementary Table S5. After data processing, an average of 28.4 million clean paired-end reads were acquired for each sample. When we mapped these reads onto the human GRCH38 version 23 (Ensembl 81) genome using STAR, 87.03% and 89.83% of the sequences in the two experimental groups were identical, while only 65.17% of the sequences in the control group were identical (Supplementary Table S6). Most unaligned reads resulted from the presence of broken adaptors and broken primer sequences. We recovered 2,839,338 and 1,632,085 uniquely located readings from the two IP groups for deeper analysis in this study.

**Figure 3:**
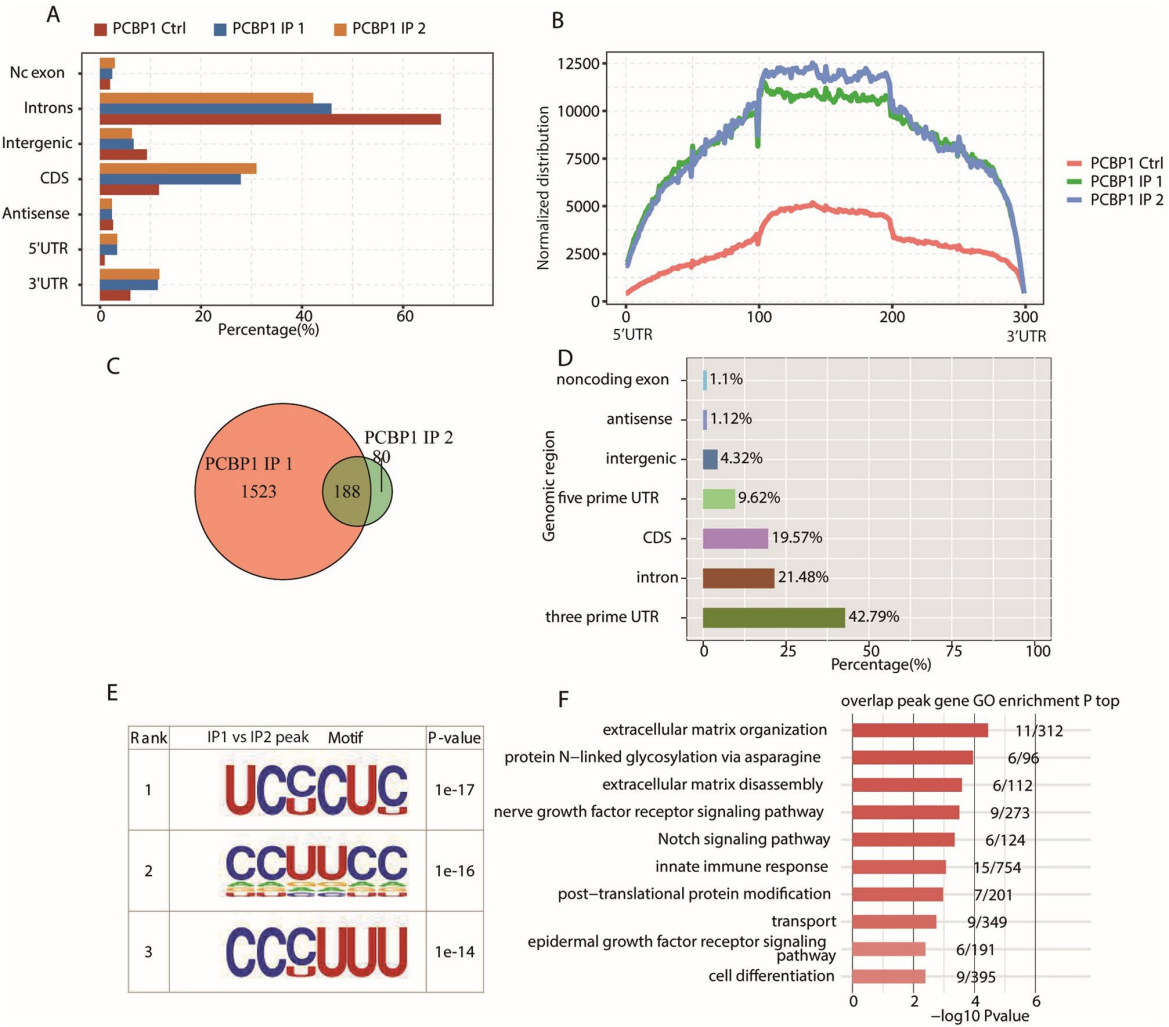
Analysis of binding characteristics of PCBP1. (**A**) Bar plot showing the reads distribution across different genomic regions. (**B**) Peak reads density in 5′UTR, CDS and 3′UTR. These three regions of each gene were separated into 100 bins, the PCBP1 peak reads in each bin was calculated. The reads density of PCBP1 peaks in all genes were plotted. (**C**) Venn diagram showing the overlapped peaks in two IP samples. (**D**) Bar plot showing the distribution of overlapped peaks across different genomic regions. (**E**) Motif analysis showing the top 3 preferred bound motifs of PCBP1 using HOMER software. (**F**) Bar plot exhibited the most enriched GO biological process results of PCBP1-bound genes. PCBP1, Poly C Binding Protein 1; UTR, untranslated region.

Ribonuclease is usually used to digest immunoprecipitated RNAs in RBP-RNA complexes in CLIP-seq protocols to generate short-length reads from sites protected by RBPs^28^. The results of eCLIP-seq reads were compared with the distribution ratios of different regions in the genome. Compared with those in the control group, the reads in the IP groups were mainly enriched in the CDS, 3′UTR and 5′UTR (Fig. 3A). When analysing the coverage and characteristics of the genome, we divided the 5′UTR, CDS, and 3′UTR of the gene into 100 copies, each called a bin, and then we calculated the sum of the numbers of the average reads in each bin to obtain the overall read coverage of each bin. Our results showed that the enrichment of the IP groups was significantly higher than that of the control group (Fig. 3B), which indicated that PCBP1 was relatively enriched in the gene body.

After removing the gene interval peak and intron overlap peak from the binding peak obtained in the previous step, ABLIRC (ABlife) was used for peak calling. We identified 1711 and 268 peaks in the two IP groups. A Venn diagram showed that there were 188 peaks shared by the two IP groups (Fig. 3C), which affirmed the high repeatability of the two IP experimental groups and the high reliability of the results. Then, we used a similar method to map 188 peak clusters to the human genome to observe their distribution in different regions of the genome. The results show that the peaks are mainly distributed in the 3'UTR, intron and CDS regions (Fig. 3D). RBP binding in the 3'UTR often affects mRNA stability, and binding in the CDS region is often related to AS. We used Hypergeometric Optimization of Motif EnRichment (HOMER v4.11, http://homer.ucsd.edu/homer/) to perform motif analysis on the specific binding peaks of the experimental samples. The motif enrichment results of the peaks shared by the IP1 and IP2 groups showed that CU was enriched in the PCBP1-bound motif (Fig. 3E). We aligned the gene sequences corresponding to 188 peak clusters to the GO database for GO annotation, and the results indicated that these genes were mainly enriched in the process of tumour metastasis, such as ECM organization and ECM disassembly (Fig. 3F). This was consistent with the results of the RNA-seq analysis and the AS analysis.

### One hundred forty-five genes bind to PCBP1 and undergo AS under the regulation of PCBP1 at the same time

**Figure 4:**
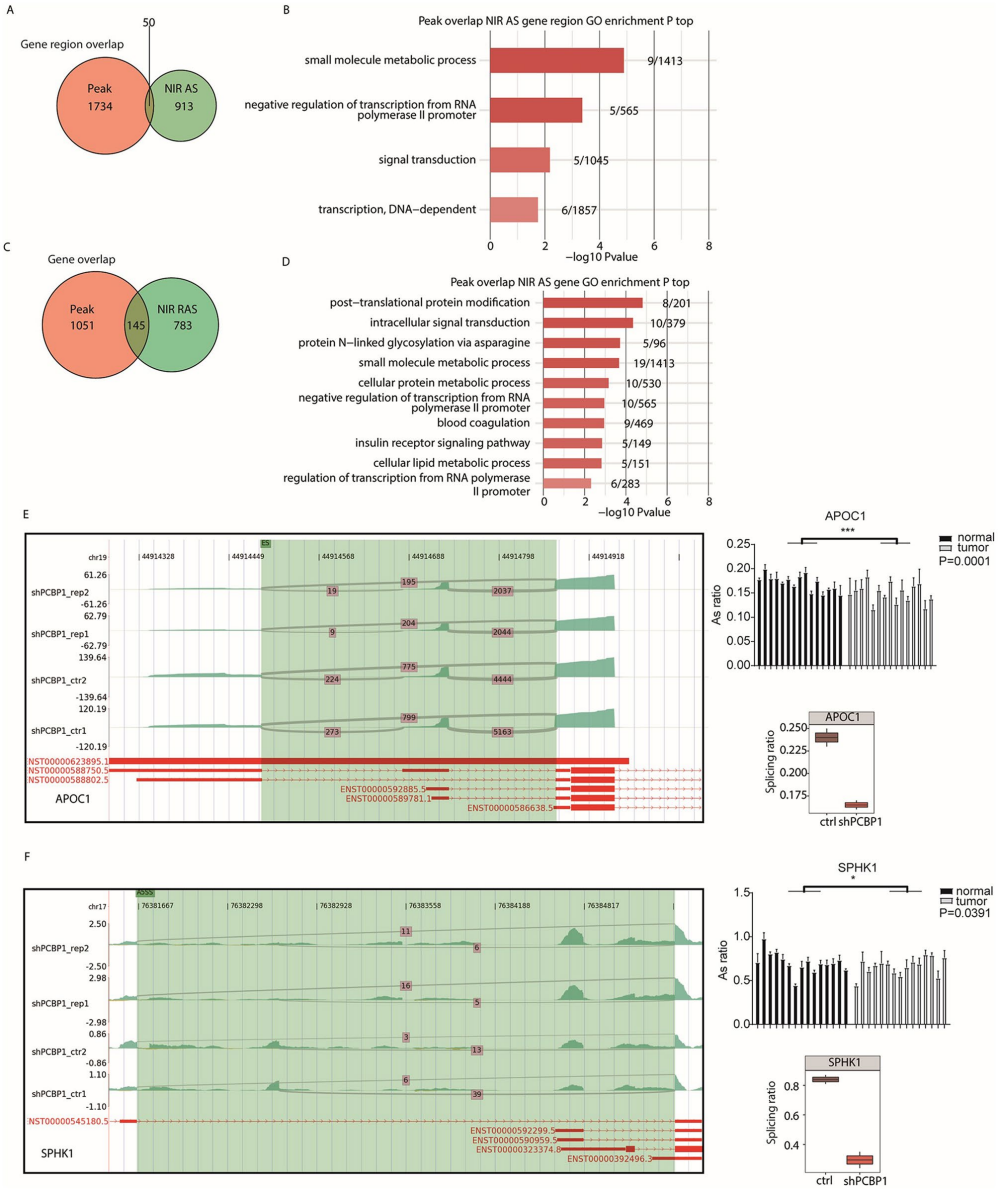
PCBP1 selectively binds to mRNA to regulate alternative splicing. (**A**) Venn diagram showing the overlap of PCBP1-bound peaks and PCBP1-regulated alternatively NIR splicing events (use NIR region), and the PCBP1-bound peaks is the union of two IP samples, bound peaks cluster (several peaks will appear together, which can be considered as one peak). (**B**) Bar plot exhibited the most enriched GO biological process results of the overlap peak genes in Fig. (**A**). (**C**) Venn diagram showing the overlap of PCBP1-bound peaks and PCBP1-regulated alternatively NIR splicing events (Use Symbol), and the PCBP1-bound peaks is the union of two IP samples. (**D**) Bar plot exhibited the most enriched GO biological process results of the overlap peak genes in Fig. (**C**). (**E**) IGV-sashimi plot showed the alternative 3′ splicing sites events (A3SS) in CELF1. Reads distribution of each alternative splicing event was plotted in the left panel with the transcripts of each gene shown below. The schematic diagrams depict the structures of ASEs. (**F**) IGV-sashimi plot showed the alternative 5’ splicing sites events (A5SS) in SPHK1. Reads distribution of each alternative splicing event was plotted in the left panel with the transcripts of each gene shown below. The schematic diagrams depict the structures of ASEs. PCBP1, Poly C Binding Protein 1; mRNA, messenger ribonucleic acid; NIR, non-intron-retention regulated; GO, gene ontology. IGV, The Integrative Genomics Viewer.

We performed an association analysis between the peak genes shared by the two IP groups through eCLIP-seq analysis and AS analysis, and the results showed that 50 gene regions where AS occurred at areas where PCBP1 would bind (Fig. 4A). We performed GO functional enrichment analysis on the obtained genes with AS at the common binding position, and the results showed that the functions of these overlapping regions were most enriched in biological processes that are involved in the negative regulation of transcription from the RNA polymerase II promoter, small molecule metabolic process, signal transduction and transcription (Fig. 4B). Similarly, we identified a total of 145 genes that bind to PCBP1 and undergo AS under the regulation of PCBP1 at the same time (Fig. 4C). Among the top 10 most enriched GO biological process terms (Fig. 4D), posttranslational protein modification, cellular lipid metabolic process and regulation of transcription from the RNA polymerase II promoter were closely related to malignant tumour biological behaviour. The enriched KEGG pathways of the overlapping gene regions and genes (*P* > 0.05) included those involved in bacterial invasion of epithelial cells, protein processing in endoplasmic reticulum and Vibrio cholerae infection, and endocrine resistance (Supplementary Fig. S2A, B). The genes involved in these GO terms and KEGG pathways are presented in Supplementary Table S7.

### Further validation of PCBP1-regulated gene expression and AS

To validate the accuracy of the predicted PCBP1-regulated ASEs selected from the RNA-seq data, the 5 RASEs classified into ES, cassette exon, A5SS and A3SS were selected for q-PCR validation. All RASEs selected for verification were annotated in the KEGG analysis. We present PCR primer pairs in Supplementary Table S8, and the primers were designed to amplify the long splicing isoforms and the short splicing isoforms in one reaction. For several of which were selected, the verification results of APOC1, SPHK1 and IP6K2 showed significant differences. The probability of the ASE IP6K2 is downregulated, contrary to expectations. The q-PCR results validated these two ASEs, which were confirmed to play a key role in tumour metastasis and were located in the APOC1 (*P* = 0.0001) and SPHK1 genes (*P* = 0.0391) (Fig. 4E and F). In summary, our experiments confirmed that our findings on PCBP1-regulated gene expression and ASEs are convincing.

### PCBP1 expression is different in HCC and normal tissues and is associated with clinical features of HCC

**Figure 5:**
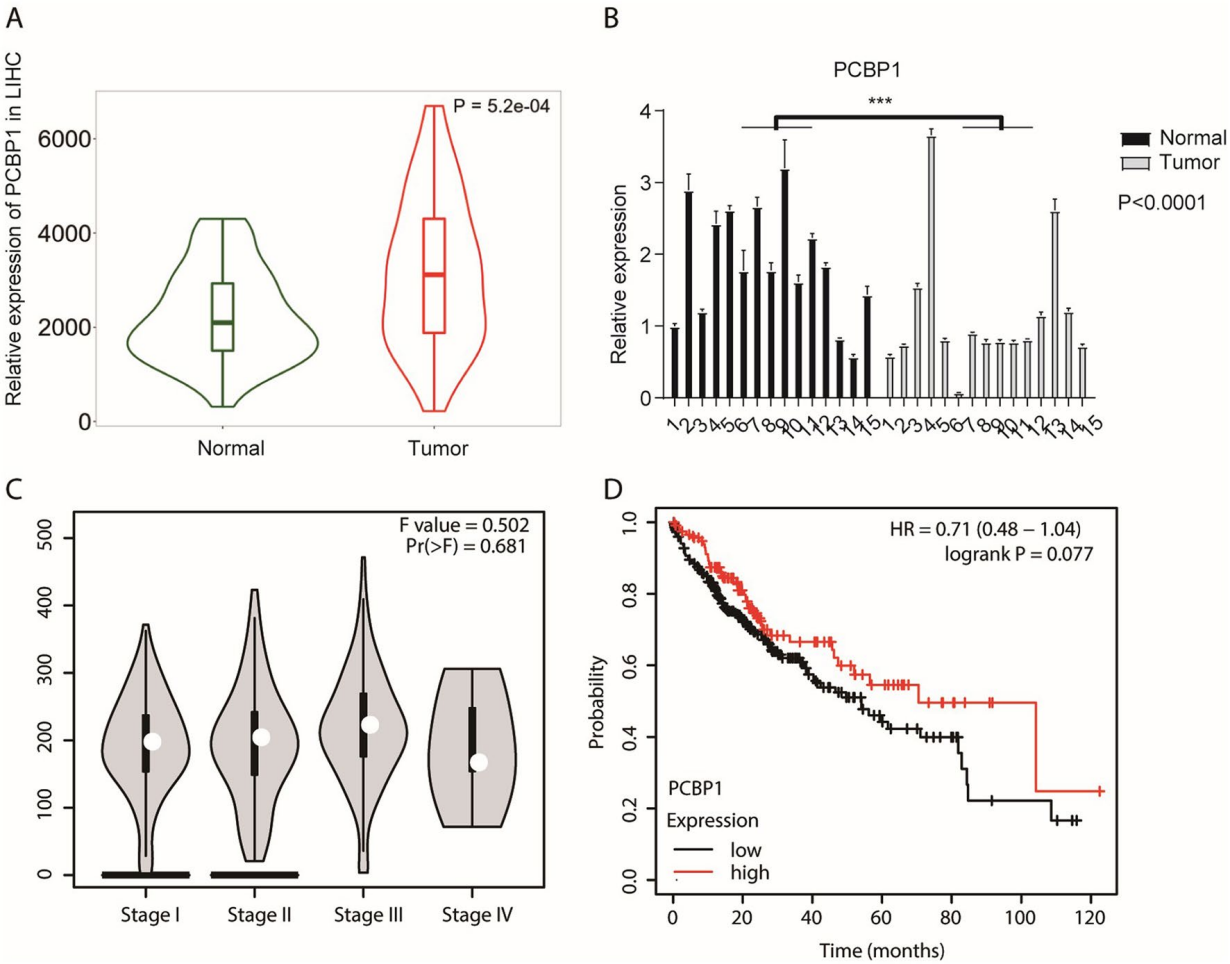
The expression level of PCBP1 in HCC tissues was lower than that in normal tissues, and the survival time of patients with low expression of PCBP1 was lower. (**A**) Based on liver hepatocellular carcinoma (HCC) data in TCGA database, the expression level of PCBP1 in HCC tissues was higher than that in normal tissues. (**B**) Quantitative experiments were carried out in 15 cases of HCC and 15 cases of normal liver tissues. The results showed that the expression level of PCBP1 in HCC tissues were lower than that in normal tissues, that is, the expression level of PCBP1 was downregulated. (**C**) Based on the data of HCC in TCGA database, the expression level of PCBP1 in different stages of HCC was analyzed. (**D**) Prognostic analysis of PCBP1 was performed using the kmplot online tool, and the results showed that the survival time of patients with low expression of PCBP1 was lower. PCBP1, Poly C Binding Protein 1; HCC, hepatocellular carcinoma; TCGA, the cancer genome atlas program.

To verify the reliability of the PCBP1 gene expression difference between the experimental groups and the control groups retrieved from the ENCODE database in this study, we searched The Cancer Genome Atlas (TCGA) database and drew a PCBP1 expression level distribution map based on the HCC data from the TCGA database. We found that the expression level of PCBP1 in tumour tissues was higher than that in adjacent tissues (*P* = 5.2e−04) (Fig. 5A). To confirm the expression level difference of the PCBP1 gene between cancer tissues and normal tissues, q-PCR was conducted, and the results showed that PCBP1 gene expression in 15 patients with HCC was decreased compared with that in 15 samples of cancer tissue (*P* < 0.0001). Two-way ANOVA was performed to confirm the significant difference between the two groups. However, the results of RT-qPCR experiments conducted on 15 samples of HCC and 15 samples of normal liver tissues were contrary to the above conclusions (Fig. 5B). The experimental results showed that the expression level of PCBP1 in HCC tissues was lower than that in normal tissues (*P* < 0.0001). Previous studies have shown that RBPs are significantly related to the clinical stage and prognosis of tumours. This difference might be due to the different clinical stages of HCC. Tumour diameter and degree of differentiation may also cause this phenomenon. Based on the HCC data in the TCGA database, we showed the expression level of PCBP1 in different stages of HCC (Fig. 5C). To further investigate the correlation between PCBP1 and clinical forecasting in HCC patients, we evaluated the prognostic implications of PCBP1 using the TCGA database (http://www.oncolnc.org/). We used the “Auto select best cutoff” option for automatic calculation, and the final expression cutoff used is 15,686. The p value does not reach the general threshold of 0.05, but it is still significant from the graph. We speculated that patients with lower PCBP1 expression levels have a poorer prognosis. The KM Plotter online tool was used for prognostic analysis to confirm this hypothesis. The results showed that patients with low PCBP1 expression had a shorter survival time (Fig. 5D).

## Discussion

As a feature-rich RBP, PCBP1 participates in mRNA metabolism regulation^16^, and alterations in PCBP1 function accelerate tumour metastasis and progression^29^. In addition, various lines of evidence have proven that PCBP1 is linked with the prognosis of carcinoma^15^. In SH-SY5Y cells, Huo et al. knocked down endogenous PCBP1, and then identified 328 upregulated transcripts and 47 downregulated transcripts through microchips^30^. Among these transcripts, the signaling of a ligand–receptor relationship between SEMA6A and plexin-A2 can regulate cell migration^31^. Growth differentiation factor 15 (GDF15) is associated with tumorigenesis^32^. The differential expression profile of this study shows that PCBP1 is widely involved in the regulation of transcripts. In our research, we revealed that after PCBP1 knockdown, the expression of a large number of metabolic process-related genes was downregulated, while the expression of genes related to cell metastasis was upregulated. The results of RT-PCR and sequencing of HCC tissues indicated that PCBP1 expression was downregulated, suggesting that PCBP1 is a TSG in HCC. The PCBP1 gene difference between the experimental groups and the control groups retrieved from the ENCODE database and verified by q-PCR experiments are different, which may be due to the small sample size. Through data analysis of genome‑wide transcription and ASEs in the PCBP1 knockdown group and control group utilizing RNA‑seq data from HepG2 cells, it was revealed that PCBP1-binding target genes are enriched in metastasis-related pathways, which are consistent with the upregulated gene functions. In a variety of tumour cell lines, PCBP1 promotes the transformation of oncogenic subtypes to tumour suppressor subtypes by inhibiting the alternative splicing of an important intermediate molecule, thereby affecting tumour invasion. Wang et al. overexpressed 26 splicing factors in HEK293 cells and analysed the alternative splicing of STAT3 exon 23. Their results showed that PCBP1 regulates the alternative splicing of STAT3 exon 23 to promote the transformation of oncogenic subtype STAT3α to the tumour suppressor subtype STAT3β^14^. Moreover, PCBP1 affects the alternative splicing of integrin β1, promotes the transformation of integrin β1 subtype A to subtype C in pancreatic cancer cells, and inhibits the metastasis of pancreatic cancer cells in this way^13,33^. Our results also indicated that after PCBP1 expression levels changed, the abnormally spliced genes were related to metabolic processes and apoptosis processes and may be involved in metastasis. These findings indicate that the relationship between PCBP1 and the biological behaviour of tumour metastasis may be more diverse than previously recognized.

We comprehensively analysed DEGs whose expression level was regulated by PCBP1. We found that these genes were enriched in multiple GO functional clusters and KEGG pathways. The regulated genes after PCBP1 depletion were enriched in ECM organization and cell adhesion. The metabolism of ECM is out of control in tumours, while remodelling and sclerosis of the ECM are important characteristics of tumours^34,35^. In particular, the importance of ECM remodelling in cancer has been recognized. Previous studies have found that the induction of collagen cross-linking makes the ECM harder, promotes focal adhesion, and induces the invasion of epithelial cells initiated by oncogenes^19,34,36^. Collagen remodelling could create space for cell migration^37^. In fact, ample evidence has revealed that abnormal tumour cell adhesion deeply contributes to tumour metastasis and progression. A broad array of adhesion molecules, such as cadherins and CAMs, has been found to play important roles in tumour malignancy and metastasis.

The expression level of E-cadherin, the prototypic epithelial adhesion molecule that functions in adherens junctions, is downregulated in most epithelial cancers. Previous studies have shown that reconstitution of the functional E-cadherin adhesion complex restrains the invasiveness of many types of tumours^38–40^. PCBP1 also regulates the AS of a large number of genes. These genes were mainly enriched in small molecule metabolic processes, cellular protein metabolic processes and cellular lipid metabolic processes. Studies of human primary and metastatic tumour tissue samples have shown that the metabolism of many small molecules is associated with the metastasis of tumours^41–43^. For example, the expression of the small-molecule inhibitor CKB is enhanced in liver metastases and inhibits extracellular small molecule metabolism. This may be a key way to control the metastasis of colon cancer to the liver. In vitro and in vivo, the activity of the Jumonji family of histone demethylases is especially inhibited by the small molecule JIB-04. In mice, JIB-04 reduced the tumour burden and prolonged survival time. The small molecule FPR1 is expressed in different types of tumours and plays an important role in tumour expansion resistance and recurrence.

Among the five genes we identified that were bound at the splicing site, the results of APOC1 and SPHK1 verified by RT-qPCR in HCC tissues were consistent with our predictions. Decreased PCBP1 expression might lead to the abnormal splicing of these two genes, thereby promoting HCC metastasis. Interestingly, APOC1 is a special member of the apolipoprotein family that has multiple functions. It could not only transport lipids but also regulate pathological processes such as diabetes, Alzheimer’s disease and inflammation^44–47^. Previous studies speculated that APOC1 may act as a promoter in the progression and development of various cancers^48–52^. For example, the relative expression of APOC1 in cervical tumour tissues was obviously higher than that in adjacent nontumour tissues (*P* < 0.05). Silencing of APOC1 restrained cell progression and EMT in cervical cancer cell lines, while APOC1 overexpression remarkably accelerated tumour progression and EMT (*P* < 0.05). In vivo and in vitro, the overexpression of APOC1 accelerated cell progression and EMT (*P* < 0.05)^53^. Additionally, APOC1 expression was distinctly higher in gastric cancer and had a close relationship with some tumour prognostic indicators, such as clinical stage, tumour classification, lymph node metastasis and shorter overall survival and relapse-free survival^48^. In gastric cancer, abnormal expression of APOC1 can predict the clinical characteristics and unfavourable prognosis of patients^48^. However, there are few studies on APOC1 in HCC, revealing that the molecular regulation mechanism of APOC1 in HCC may be a potential research direction. As an important regulator of lipid metabolism, SPHK1 (sphingosine kinase 1) plays a causal role in promoting malignant tumour biological properties through multiple pathways. SPHK1 regulates the PTK2/FAK (protein tyrosine kinase 2) pathway and activates the EGFR (epidermal growth factor receptor) pathway to confer tumour metastasis in colon cancer and oesophageal cancer^54,55^. SPHK1 protein expression and mRNA transcription are both upregulated in HCC tissues compared with adjacent non-HCC tissues^56^. The epithelial marker CDH1 was downregulated when SPHK1 was overexpressed, some tumour-related metabolic processes, such as cell invasion and migration, were promoted, and the EMT process was induced. EMT was also activated when SPHK1 accelerated the lysosomal degradation of CDH1 depending on TRAF2 (TNF receptor-associated factor 2)-mediated macrophage activation^57^. This suggested that blocking SPHK1 activity to attenuate autophagy may be a promising strategy to prevent and treat HCC. In this study, RNA-seq was successfully applied to research the role of PCBP1 in a human HCC cell line, and we also demonstrated the key role of PCBP1 in regulating transcription and AS. We showed that PCBP1 regulated the transcription of genes involved in metastasis-related functions, including ECM organization and cell adhesion. Interestingly, PCBP1 also affected the AS of genes enriched in small molecule metabolic processes, which play an important role in tumour expansion and recurrence. It also regulates the AS of the APOC1 and SPHK1 genes, whose functions are associated with tumour metastasis and tumorigenesis. Our research presented several molecular mechanisms and revealed the regulatory role of PCBP1 in influencing HCC metabolism in physiological and pathological states.

## Methods

### Retrieval and processing of public data

Public sequence data files were obtained from the ENCODE database (https://www.encodeproject.org/). The FASTX-Toolkit (v.0.0.13; http://hannonlab.cshl.edu/fastx_toolkit/) was used to eliminate low-quality bases from the raw reads. Then, the clean reads were appraised by the quality control tool FastQC (http://www.bioinformatics.babraham.ac.uk/projects/fastqc).

### Read alignment and differentially expressed gene (DEG) analysis

HISAT2 was used to make clean reads align with the human GRCh38 genome^58^. The read numbers and fragments per kilobase of exon per million fragments mapped (FPKM) values of each gene were calculated using uniquely mapped reads. The gene expression level was estimated using FPKM values. The software DESeq2^59^, which is usually used to study differential gene expression, was applied to screen the RNA sequencing (RNA-seq) results for DEGs. When determining whether a gene was differentially expressed, the results were analysed based on the fold change (fold change ≥ 2 or ≤ 0.5) and false discovery rate (FDR < 0.05).

### Alternative splicing (AS) analysis

We characterized and appraised the alternative splicing events (ASEs) and regulated alternative splicing events (RASEs) between the samples using the ABLas pipeline^60,61^. ASEs were detected based on the reads of splice junctions. The ten kinds of ASEs included exon skipping (ES), alternative 5′ splice site (A5SS), alternative 3' splice site (A3SS), intron retention (IR), mutually exclusive exons (MXE), mutually exclusive 5' UTRs (5pMXE), mutually exclusive 3'UTRs (3pMXE), cassette exon, A3SS&ES and A5SS&ES. When comparing samples, the alternative reads and model reads of the sample were selected as input data. We calculated the changed ratio between alternately spliced reads and constituted spliced reads between comparison samples, which was also defined as the RASE ratio. We set RASE ratio >  = 0.2 and p-value <  = 0.05 as the thresholds for RASE detection. For repetition comparisons, Student’s t-test was implemented to check the significance of the ratio alteration of ASEs. If the events were significant at a P-value cut-off of 0.05, they were considered RASEs.

### Enhanced crosslinking and immunoprecipitation sequencing (eCLIP) analysis

The modified individual nucleotide resolution eCLIP protocol was applied to map the RNA-binding protein (RBP) binding sites on their target RNAs. This process observably improves the efficiency and decreases the execution complexity. This method significantly minimizes the amplification bias by the connection of barcoded single-stranded DNA adapters.

First, RNAs and the target proteins are UV-crosslinked, followed by lysing cells and treating lysate. Second, the complexes of protein-RNA are immunoprecipitated and linked to an RNA adapter at the 3' end of the target RNA. Proteinase K is used to digest and eliminate the bound protein before RNA reverse transcription. The ultimately acquired cDNA is ligated to the 3' end of single-stranded DNA adapters. The adapters contain either an N5 or N10 sequence serving as a unique identifier against PCR duplicates. The final step is to amplify and sequence the paired-ended cDNA fragment.

### Functional enrichment analysis

We performed Gene Ontology (GO) and Kyoto Encyclopedia of Genes and Genomes (KEGG) pathway enrichment analyses using the KOBAS 2.0 server to ascertain the functions of the genes and obtain the functional category distribution frequency^62^. We utilized the hypergeometric test together with the Benjamini–Hochberg FDR-controlling protocol to characterize the enrichment of every term. Reactome (https://reactome.org/) provides abundant pathway annotation information, so we used it to analyse the functional enrichment of the sets of selected genes.

### Tissue specimens

All HCC specimens were obtained from patients who underwent surgical resection of their diseases and provided informed consent before surgery on their liver. The cancer and normal tissues taken from resected specimens were immediately frozen at − 80 °C until RNA extraction. Both tumour and adjacent nontumour tissues were sampled, with approximately 1 cm3 of each specimen, and were proven by pathological examination. This study was approved by the Ethics Committee of The First Affiliated Hospital of Zhengzhou University.

### Gene expression microarray data analysis

Gene expression data were retrieved from the Gene Expression Omnibus database (https://www.ncbi.nlm.nih.gov/geo). The microarray data probe was transformed to gene symbols. For multiple probes that mapped to one gene symbol, the average value was considered the final expression result of this gene. The online GEO2R tool with default parameters was used (https://www.ncbi.nlm.nih.gov/geo/geo2r/) to compare two or more groups of samples to identify genes that are differentially expressed under experimental conditions.

### Reverse transcription qPCR validation of PCBP1 gene expression and ASEs

To verify the validity of the expression difference of the PCBP1 gene and ASEs in HepG2 cells, we performed quantitative reverse-transcription polymerase chain reaction (RT-qPCR) for some RASEs of interest. The primers used for RT-qPCR were normalized to the reference gene human GAPDH. RT-qPCR was carried out by a StepOne Real-Time PCR System by QuantStudio 6 (Applied Biosystems Co., Ltd.). The PCR procedures were performed with the following steps: the samples were first denatured at 95 °C for 5 min, followed by 40 cycles of denaturation at 95 °C for 10 s and then annealed and extended at 60 °C for 30 s. PCR amplification was repeated three times for normal and HCC samples.

### Prognosis and expression level analyses

The KM Plotter online tool was used for prognostic analysis with the default parameters (URL: http://kmplot.com/analysis/index.php?p=service&cancer=liver_rnaseq. GEPIA2's box plot (http://gepia2.cancer-pku.cn/#analysis) and TNMplot (https://www.tnmplot.com/) were used to visualize the expression of PCBP1 in normal tissues and HCC tissues, and the violin chart of TNMplot was displayed. We used the stage plot of GEPIA2 (http://gepia2.cancer-pku.cn/#analysis) to compare the expression of PCBP1 in HCC tissues in different stages.

### Other statistical analysis

The data were analysed using R software (v4.0.1, https://www.r-project.org/). The R package factoextra (https://cloud.r-project.org/package=factoextra) was used to analyse the principal components to show the clustering of samples with the first two components. RNA reads were normalized by the tags per million (TPM) of each gene across all samples.

After standardizing the reads by the TPM of each gene across all samples, an in-house script (sogen) was used for next-generation data sequence visualization and genomic annotations. We employed the pheatmap package (https://cran.r-project.org/web/packages/pheatmap/index.html) in R to conduct the clustering based on Euclidean distance. Student’s t-test was performed for comparisons of two different groups.

### Ethics statement

This study had gained the informed consent from each subject and been approved by the Ethics Committee of the First Affiliated Hospital of Zhengzhou University. The authors confirms that all experiments and public data acquisition were performed in accordance with relevant named guidelines and regulations.

## Supplementary Information

Supplementary Information 1.

Supplementary Information 2.

Supplementary Information 3.

## Data Availability

The original contributions presented in the study are included in the article/Supplementary Material. Public sequence data files were downloaded from the Encode project. Gene expression profile was obtained from the Gene Expression Omnibus database. Further inquiries can be directed to the corresponding author.

## Abbreviations

PCBP1: Poly C binding protein 1
RBPs: RNA binding proteins
HCC: Hepatocellular carcinoma
CLIP: Crosslinking immunoprecipitation
HBV: Hepatitis B virus
HCV: Hepatitis C virus
TSGs: Tumor suppressor genes
RNP: Ribonucleoprotein
CELF1: CUG RNA-binding protein and embryonically lethal abnormal vision-type RNA-binding protein 3-like factor 1
QKI: QUAKING RNA Binding Protein
EMT: Epithelial-mesenchymal transition
IncRNA: Long non-coding RNA
DEG: Differentially expressed gene
FPKM: Fragments per kilobase of exon per million
FDR: False discovery rate
AS: Alternative splicing
ASEs: Alternative splicing events
RASEs: Regulated alternative splicing events
ES: Exon skipping
A5SS: Alternative 5′ splice site
A3SS: Alternative 3′ splice site
IR: Intron retention
MXE: Mutually exclusive exons
5pMXE: Mutually exclusive 5′UTRs
3pMXE: Mutually exclusive 3′UTRs
cDNA: Complementary deoxyribonucleic acid
GO: Gene ontology
KEGG: Kyoto encyclopedia of genes and genomes
RT-qPCR: Quantitative reverse-transcription polymerase chain reaction
PCA: Principal component analysis
TPM: Tags per million
TNF: Tumor necrosis factor
ECM: Extracellular matrix
CAMs: Cell adhesion molecules
ANOVA: Analysis of variance
HOMER: Hypergeometric optimization of motif enrichment
APOC1: Apolipoprotein C1
SPHK1: Sphingosine kinase-1
IP6K2: The inositol hexakisphosphate kinase6
TCGA: The cancer genome atlas program
CKB: Creatine kinase, brain-type
PTK2: Protein tyrosine kinase 2
EGFR: Epidermal growth factor receptor
TRAF2: TNF receptor associated factor 2
NIR: Non-intron-retention regulated
