# Supplementary material for: PCBP1 regulates the transcription and alternative splicing of metastasis‑related genes and pathways in hepatocellular carcinoma
Source: Sci Rep. 2021 Dec 2;11:23356. doi: 10.1038/s41598-021-02642-z (PMC8640068; doi:10.1038/s41598-021-02642-z)
Supplement: Supplementary file 1 — Supplementary Information 1. [file 41598_2021_2642_MOESM1_ESM.pdf]

Supplementary Figure S1

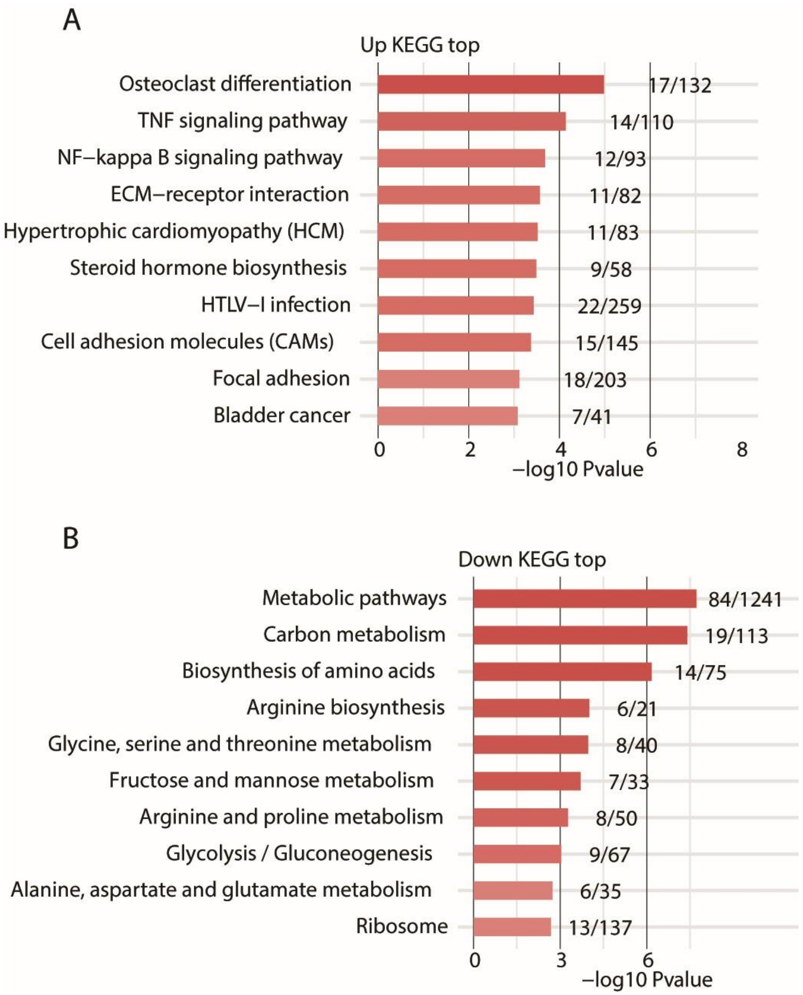

Supplementary Figure S2

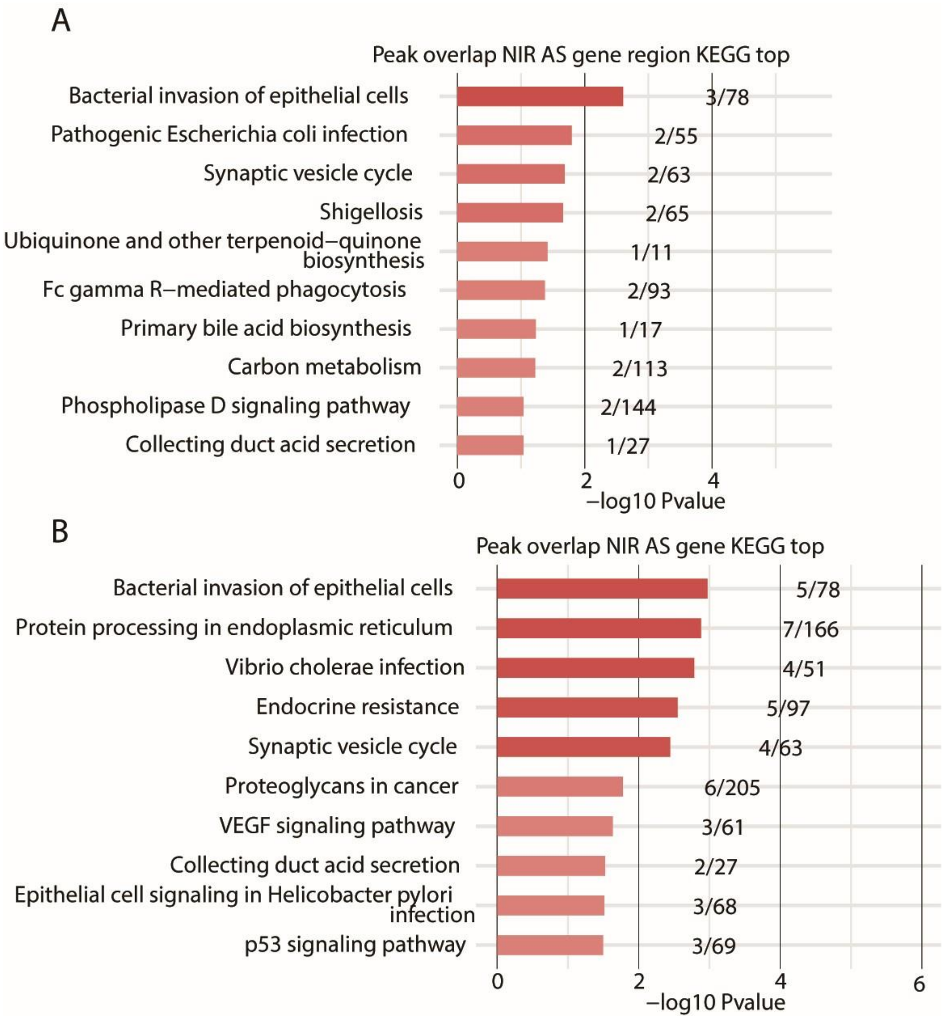

Supplementary Table S1

Summary of sample names, the RNA-seq sequencing information and mapping results in each sample

Sample	Raw	Clean	Clean Per	Raw base	Clean base	Base Per	Unique tag	Q20	Q30	GC	DUP
shPCBP1_ctr1	84407518	84407518	100.00%	8.44G	8.44G	100.00%	34073034(40.37%)	91.70%	83.89%	47.50%	79.40%
shPCBP1_ctr2	64334356	64334356	100.00%	6.43G	6.43G	100.00%	27593546(42.89%)	91.75%	84.15%	47.50%	77.08%
shPCBP1_rep1	62191090	62191090	100.00%	6.22G	6.22G	100.00%	30886454(49.66%)	91.14%	81.61%	48%	71.41%
shPCBP1_rep2	65532642	65532642	100.00%	6.55G	6.55G	100.00%	31894596(48.67%)	91.06%	81.78%	47.50%	72.33%

Supplementary Table S2

The results of reads mapped to the human genome

Sample	shPCBP1_ctr1	shPCBP1_ctr2	shPCBP1_rep1	shPCBP1_rep2
Total reads	84407518	64334356	62191090	65532642
Total mapped	80660454(95.56%)	61918932(96.25%)	60106988(96.65%)	63118870(96.32%)
Total Uniquely mapped	75564014(93.68%)	58249151(94.07%)	56779196(94.46%)	59553483(94.35%)
Total Multiple mapped	5096440(6.32%)	3669781(5.93%)	3327792(5.54%)	3565387(5.65%)
Total Pairs	42203759	32167178	31095545	32766321
Total Uniquely Concordant Pairs	36374367(86.19%)	28092252(87.33%)	27214135(87.52%)	28563000(87.17%)
Splice reads	28623772(37.88%)	21541225(36.98%)	19009539(33.48%)	19596129(32.91%)
Nonsplice reads	46940242(62.12%)	36707926(63.02%)	37769657(66.52%)	39957354(67.09%)

Supplementary Table S5

ECLIP-seq samples descriptions

Sample	Raw	Clean	Clean Per	Raw base	Clean base	Base Per	Unique tag	Q20	Q30	GC	DUP
PCBP1_IP_1	30792408	30792408	100.00%	1.32G	1.35G	102.33%	8502068(27.61%)	98.78%	96.75%	64.50%	76.50%
PCBP1_IP_2	27537400	27537400	100.00%	1.18G	1.21G	102.33%	10313776(37.45%)	98.83%	96.88%	63%	69.30%
PCBP1_Ctrl	26955178	26955178	100.00%	1.16G	1.35G	116.28%	10025357(37.19%)	98.85%	97.15%	57%	66.84%

Supplementary Table S6

The mapping results of trimmed ECLIP-seq reads on the Homo sapiens genome (GRCH38 version).

Sample	PCBP1_Ctrl	PCBP1_IP_1	PCBP1_IP_2
Total reads	26955178	30792408	27537400
Total mapped	21081102(78.21%)	21893259(71.1%)	16051714(58.29%)
Total Uniquely mapped	7341598(34.83%)	2839338(12.97%)	1632085(10.17%)
Total Multiple mapped	13739504(65.17%)	19053921(87.03%)	14419629(89.83%)
rmdup reads	4609798(62.79%)	1875243(66.05%)	1062999(65.13%)
Sense reads	4489915(97.4%)	1830712(97.63%)	1037708(97.62%)
Antisense reads	119883(2.6%)	44531(2.37%)	25291(2.38%)

Supplementary Table S7

Genes involved in each GO term or KEGG pathway

TCF25
AES
AMBRA1
FHL3
COPS7A

UTRN
PIGZ
VKORC1
KDM5C
GDPD5
GNAS
ATP6V0B
MINK1
GRB7
FBXO31
PTK2
MGAT1
SORT1
CEP170B
SEC31B
CELF1
TFR2
PLXNB1
ZMYND8
DLGAP4
RCOR3
KDM5A
FBRSL1
STK40
SUN1
HOXA3

LINC00963
PSMF1
ASPH
TCEA2
FLOT2
SLC2A8
SLC27A1
PIEZO2
PXN
DDX39A
SH2B1
MST1P2
GATSL2
NSMF
SPHK1
ARSB
ANKRD11
KIAA0319L
LRP5
9-Sep
RAB11FIP3
EIF5A
WNK4
FLNB
ACLY
HNRNPUL1

SCAMP5
SIN3A
NOL4L
CD46
RABL6
TMC6
MDH2
STAT6
TUBB3
CXXC5
ATG9A
S100A1
S100A16
PLOD2
EPN1
SPAG4
SEC31A
FAAP20
ADGRG6
HSD3B7
PTPN23
PRKCSH
ASGR2
OBSL1
IP6K2
CARM1

EXOC3L4
FOXN3
AGRN
CNPY3
NPEPPS
SLC7A5
SDF4
PABPC1L
SEC24C
AXIN2
C16orf13
CIC
MARK2
ABCC10
TSPEAR-
AS1
OS9
KIAA0195
MSI2
PLEC
EXD3
DNM2
HNF4A
TCIRG1
ARHGAP5
PLXNB2

CCDC85C
CYB5R3
CROCCP2
ARHGEF1
MAT1A
P4HA1
TPT1-AS1
ALG3
JUP
CHRD
CDKN1A
BRPF3
TKFC
GUK1
PLD3
SERF2
HDAC10
PCBP2
SREBF1
MAP4K4
PARD3
ATP6V0E2
MGRN1
SNHG14
ESPN
UBE2D2

ATXN2L
BAX
SERPINA5
DAPK1
FKBP1A
CTTN
MROH1
APOC1
ARID1A
SMEK2
SHISA5

Supplementary Table S8

PCR primer table for RASGs.

RASGs	PCR primer
PCBP1-F	CAGGTGGCAAGACAACAGTC
PCBP1-R	GGACATCTGGCGGATCTCAT
CELF1-M-F	CAAGCTGTGCCAGAAGACCC
CELF1-AS-F	AACGGCAGGAATCAGAAGACCC
CELF1-M/AS-R	GAACGGCTTTCAGATTGG
MSI2-M/AS-F	AGCGGCTTATGGACCAGTG
MSI2-AS-R	GGGTTGGAGCCTGATCCTCT
MSI2-M-R	CTATTCAGGACTGATCCTCT
APOC1-M-F	AAGGATTCAGAGTGCCCCTC
APOC1-AS-F	AGATTGAGAGAGTGCCCCTC
APOC1-M/AS-R	GAGCGACAGGAAGAGCCT

IP6K2-M-F	TGGCTTTGTCCTTTGTACTG
IP6K2-AS-F	CTGATGTCCCCTTTGTACTG
IP6K2-M/AS-R	CGCTTCAATGAGACAACCCT
SPHK1-M-F	TGGGACTACAGGTCCAGCCGC
SPHK1-AS-F	CCTGAACCAGGTCCAGCCGC
SPHK1-M/AS-R	TCCTGCCTTCAGCTCCTTATCG
